# The Drosophila agnostic Locus: Involvement in the Formation of Cognitive Defects in Williams Syndrome

**Published:** 2014

**Authors:** E. A. Nikitina, A. V. Medvedeva, G. A. Zakharov, E. V. Savvateeva-Popova

**Affiliations:** Pavlov Institute of Physiology RAS, nab. Makarova, 6, 199034, St. Petersburg, Russia; Herzen State Pedagogical University, nab. r. Moyki, 48, 191186, St. Petersburg, Russia; Saint-Petersburg State University, Universitetskaya nab., 7-9, 199034, St. Petersburg, Russia

**Keywords:** Williams syndrome, LIMK1, Drosophila, locomotor activity, learning, memory

## Abstract

The molecular basis of the pathological processes that lead to genome disorders is similar both in invertebrates and mammals. Since cognitive impairments in Williams syndrome are caused by LIMK1 hemizygosity, could the spontaneous and mutant variants of the *Drosophila limk1 *gene serve as a model for studying two diagnostic features from three distinct cognitive defects of the syndrome? These two symptoms are the disturbance of visuospatial orientation and an unusualy strong fixation on the faces of other people during pairwise interaction with a stranger. An experimental approach to the first cognitive manifestation might be an analysis of the locomotor behavior of *Drosophila *larvae involving visuospatial orientation during the exploration of the surrounding environment. An approach to tackle the second manifestation might be an analysis of the most natural ways of contact between a male and a female during courtship (the first stage of this ritual is the orientation of a male towards a female and following the female with constant fixation on the female’s image). The present study of locomotor activity and cognitive repertoire in spontaneous and mutant variants of the* Drosophil*a *agnostic *locus allows one to bridge alterations in the structure of the *limk1 *gene and behavior.

## INTRODUCTION

Over the past 20 years, Williams Syndrome has been regarded as one of the most attractive models for establishing a direct relationship between the genes, brain, and behavior [1, 2]. The syndrome results from a 1,500 kb deletion at 7q11.23, whose specific architecture predisposes to unequal recombination. The deletion affects approximately 20 genes; their hemizygosity manifests as a developmental anomaly characterized by cardiovascular problems, “elfin” facial features, and several typical neurological anomalies and cognitive characteristics [3]. LIM-kinase 1 hemizygosity (LIMK1 is the key actin-remodeling enzyme) causes cognitive impairments. They are characterized by a triad of signs: 1) a pronounced defect in visuospatial orientation; 2) a verbal linguistic defect of intermediate severity, which varies depending on the linguistic complexity of a certain culture; and 3) unusually strong gaze fixation on faces.

Experiments on higher animals are extremely expensive; hence, simple animal models are needed to explore for and test drugs capable of correcting these disorders.

Can *Drosophila melanogaster *be used for this purpose? On the one hand, the functions of human disease genes are often identified from the way mutations manifest themselves in the *Drosophila *gene when its sequence is identical to that in the human gene. On the other hand, all the genes that reside in mammals in a single critical region being deleted in Williams syndrome are known in *Drosophila *(let us remind readers that the *frizzled-9 *gene was first described in* Drosophila*). Despite the different evolutionary organization of the *Drosophila *genome when these genes localize on different chromosomes, the effect of a certain gene in the emergence of Williams syndrome can be analyzed if it meets the following criteria: 1) mutations of this gene must be known, while their hemizygosity would cause a mutant phenotype in *Drosophila*; 2) the architecture of the *Drosophila *gene locus may predispose to the emergence of chromosomal rearrangements due to unequal recombination; and 3) the gene locus must be characterized by increased recombination frequency, which may result in spontaneous generation of deletions or other rearrangements. This effect must manifest itself as a polymorphism in wild-type stocks that is specific to this region. The *agnostic *locus carrying the gene encoding LIMK1, which has been detected and characterized by us, meets all these criteria.

The *agnostic *locus was found in the 11 B of the X chromosome using targeted gene screening of temperature- sensitive (*ts*) mutations induced by ethyl methanesulfonate (EMS), which had the potential to inhibit the activity of enzymes of cAMP synthesis and decay [4]. Mutant *agnts3 *fruit flies exhibit an unusually high activity of Ca^2+^/calmodulin-dependent phosphodiesterase 1 [5]. Flies with a heterozygous *Df(1)368 *deletion (exposing this locus) and *Df(1)112 *deletion (isolated according to the trait of its lethality when combined with *agnts3*) also die during development at 29°C; i.e., the mutant phenotype manifests itself in hemizygous individuals (similar to that in Williams syndrome). Molecular genetic studies have showed that the *agnostic* gene encodes LIMK1 enzyme containing a repeat of two LIM domains flanked by extensive AT-rich repeats (The National Center for Biotechnology Information, NC BI). Unequal recombination is observed in this region more frequently, causing a strongly pronounced polymorphism in the wild-type stocks *Canton-S *(*CS*),* Berlin, *and *Oregon-R *(*Or-R*) [6-8].

Thus, due to its structure and nucleotide environment, the *agnostic *gene may act as a genetic reserve of polymorphism and can be used as a convenient model for genomic disorders, such as Williams syndrome. If this is true, can this model contribute to the analysis of two diagnostic symptoms of the triad of cognitive impairments in patients with Williams syndrome (disturbance of visuospatial orientation and unusual strong fixation on the faces of other people during pairwise interaction with strangers)?

The former question can be answered by analyzing the locomotor behavior in larvae, which simultaneously includes the exploration of the environment (inevitably involving visuospatial orientation) and larvae feeding (achieved when larvae move over the substrate). The latter problem can also be solved by analyzing the natural contact between an adult male and an adult female during the sexual ritual. The first stage of this ritual is the orientation of a male towards a female and following the female with constant fixation on the female’s image

In this study, we showed the changes in the parameters of locomotor activity in larvae and abrupt alterations in tracks during spatial orientation in *Oregon-R* and *agnts3 *males. Imagoes of these stocks have significant learning and memory defects caused by a conditioned reflex suppression of courtship due to abruptly enhanced orientation towards a partner and following the partner.

## EXPERIMENTAL

**Drosophila stocks**

We used stocks exhibiting polymorphism in the *agnostic* locus (region 11B of the X chromosome).

1. Wild-type stock *Canton-S *(*CS*), with temperature- sensitive (ts) mutation in the *agnostic *locus *(agnts3*) maintained in its genetic background.

2. Wild-type stock *Berlin *isolated from the natural Berlin population of *Drosophila melanogaster *and with a significant disturbance in the regulation of the *limk1* gene.

3. Wild-type stock *Oregon-R *(*Or-R*). PCR mapping of the *limk1 *gene reveals a deleted fragment between the primers limiting the region containing both LIM domains and a portion of the PDZ domain.

4. *agn^ts3^*, mutant in the *agnostic *locus containing the* limk1 *gene, carrying a 1.7 kb insertion located approximately 1 kb away from the 3’-untransribed region of the *limk1 *gene in the region where the A/T-rich sequence localizes.

**Studying the locomotor activity of larvae**

The locomotor behavior of *Drosophila *larvae was studied using an original automated construction designed by G.A. Zakharov and T.L. Payalina (Pavlov Institute of Physiology, Russian Academy of Sciences) based on a setup for recording the locomotor behavior of *Drosophila* imagoes which was designed by N.G. Kamyshev* et al*. [9]. Round cameras 20 mm in diameter were used to record the locomotor behavior of larvae. Translocation of a larva was recorded using a Logitech Quick- Cam camera. To perform automated registration of the behavior, we used the original software developed by G.A. Zakharov and N.G. Kamyshev. The experiment duration was 1 h; the temperature in the chambers was 23–24°C.

In order to analyze the rest and motion periods, the total record was subdivided into quanta 1s long. The speed of a larva at this quantum was then calculated. If the resulting speed was lower than the threshold value (0.5 mm/s), it was assumed that the larva was resting during this quantum; otherwise, the larva was assumed to be moving. The neighboring quanta with the identical type of motion were combined, thus forming motion and rest periods.

In order to analyze the dynamics of locomotor activity parameters, the total record time was divided into 300s long intervals. Each rest or motion period was considered to belong to an interval at which it had started. The locomotor frequency (number of initiated locomotions per 100s) and the activity index (share of time spent moving) were also calculated for each interval. At least 25 larvae from each stock were analyzed. The statistical significance of the differences between the experimental groups was determined using a Kruskal- Wallis dispersion analysis, followed by multiple comparisons of the mean ranks for all the experimental groups. Track distribution was compared using the paired *t*-test for the portions. The statistical significance of all differences was calculated for *p * < 0.05.

**Assessment of the learning and memorization ability**

In order to develop the conditioned reflex suppression of courtship (CR SC), a 5-day-old sexually inexperienced* Drosophila *male from the tested stock was placed in an experimental organic glass chamber (15 mm in diameter) with a fertilized 5-day-old *Canton-S *female and left for 30 min (training). Learning and memory abilities were tested immediately (0 min) and 3 h (180 min) after the training using new fertilized 5-day-old *Canton- S *females. Sexually inexperienced (naive) males were used as a control. An ethogram of male’s behavior was recorded for 300s the time when certain courtship elements (orientation, vibration, licking, a copulation attempt), as well as those not related to courtship (locomotion, preening, rest), had been started were written down. Recording was started 45s after a fruit fly was placed in the chamber. Specialized software (developed by N.G. Kamyshev) was used to decipher and analyze the data. The courtship index (CI) was calculated for each male; i.e., the time a male spent in courtship shown as a percentage of the total observation time. To qualitatively assess the learning results, we calculated the learning index (LI) using the formula:

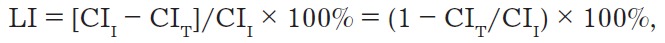



where CII and CIT are the mean courtship indices in independent samples of sexually inexperienced males and males who had been trained. Statistical processing of the results was conducted using a randomization analysis [10-12].

## RESULTS AND DISCUSSION

**Total differences in locomotor activity parameters**

**Fig. 1:**
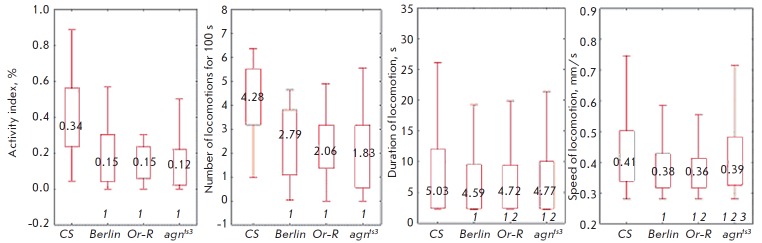
Total parameters of locomotor activity in larvae. 1 – significant difference from *CS*, 2 – significant difference from* Berlin*, 3 – *agn^ts3^*significant difference from *Or-R*. Kruskal–Wallis one-way analysis of variance with the subsequent multiple comparisons of average ranks for all experimental groups, *p* < 0.05;

The activity index, shown in *Fig. 1*, is the most general parameter describing the locomotor activity of larvae.

*CS *has a higher activity index (0.34) compared with the other stocks. One can see it from the median value and from both quartiles. No statistically significant differences between the stocks *agn^ts3^*, *Berlin, *and *Or-R* with respect to this parameter have been revealed. The activity index can be changed due to alterations in the locomotor frequency and duration. The interlinear differences in locomotion frequency are completely identical to those in the activity index. This means that changes in the activity index can be generally attributed to a decrease in locomotion frequency; however, locomotion duration can differ as well.

*CS *also differs from the rest of the stocks by this parameter.* CS *is characterized by a higher mean locomotion duration. Furthermore, *Berlin *differs from *Or-R* and *agn^ts3^*, which was not observed when examining the activity index.

Speed of locomotion is another parameter; it is independent of the previously discussed ones. The highest speed of locomotion was also observed in *CS *larvae.* agn^ts3^*occupies the second position and is followed by* Berlin *and *Or-R*. Both quartiles have the same distribution.

**Temporal dynamics of locomotor activity parameters**

**Fig. 2:**
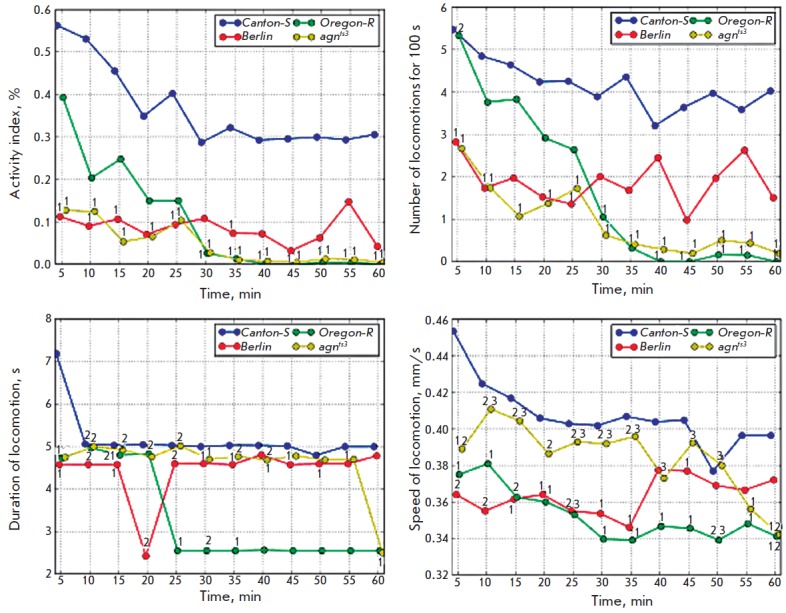
Temporal dynamics of activity index and speed of locomotion in larvae. Points show the medians of distributions. 1 – significant difference from *CS*, 2 – significant difference from *Berlin*, 3 – *agnts3 *significant difference from *Or-R*. Kruskal–Wallis one-way analysis of variance with the subsequent multiple comparisons of average ranks for all experimental groups, *p * < 0.05

The temporal dependence of locomotor activity parameters is shown in *Fig. 2*. *CS* larvae actively move immediately after they are placed in experimental chambers. The median value of the activity index is ~ 0.55. Activity subsequently gradually decreases. Starting with the 40^th^ minute, the median value of the activity index is 0.3 and further remains unchanged.

*Berlin *and *agn^ts3^*larvae under normal conditions are characterized by a considerably lower mobility compared with *CS *larvae. The median activity index is 0.1–0.15. The differences are significant up to the 25^th^ minute of the experiment and on the 35^th^ minute. After that, the differences disappear due to a drop in the activity of *CS *larvae. Mutant *agn^ts3^*are characterized by an even lower activity; the differences from *CS *are statistically significant throughout the entire experiment.* Or-R *originally has a lower mobility compared to that of *CS*, although it is higher than that in *Berlin *and* agn^ts3^*. The activity of *Or-R *larvae rapidly drops during the experiment; starting with the 30^th^ minute, it significantly differs from the activity of *CS *larvae.

Now let us thoroughly discuss the parameters contributing to the alteration of the activity index. The dynamics of the locomotion frequency are similar to the dynamics of the activity index. *CS *is characterized by an appreciably high locomotion frequency (the median value is ~ 5.5 × 10^-2^ Hz), which gradually decreases to reach ~ 3.6 × 10^-2^ Hz by the end of the experiment. *Or-R* at the beginning of the experiment has the same locomotion frequency as *CS *does (by the 5^th^ minute of the experiment it is statistically higher than that of *Berlin* and *agn^ts3^*). The locomotion frequency rapidly decreases and becomes lower than that in *CS *by the 30^th^ minute of the experiment. *Agn^ts3^*is characterized by a lower locomotion frequency than *CS *throughout the entire experiment. *Berlin *originally has a lower locomotion frequency than *CS*; however, these differences disappear after the 25^th^ minute.

Thus, *agn^ts3^*is characterized by the greatest defects compared to the *CS*. There is also a difference in the dynamics of the activities of *Berlin *and *Or-R*. The lower mobility of *Or-R *is related to a rapid decrease in activity during the experiment. *Berlin *originally exhibits a lower activity. However, it decreases slower than that in *CS; *that is why the statistically significant differences disappear in the second half of the experiment.

The dynamics of locomotion duration were rather interesting. During the entire experiment, *CS *flies (except for the first 5 min) have a virtually constant locomotion duration (5 s). *Berlin *is also characterized by a constant locomotion duration (4.5 s; the differences are statistically significant until the 55^th^ minute). The original locomotion duration of *Or-R *is identical to that of *CS *and significantly higher than that of *Berlin *. In the range between the 20^th^ and 25^th^ minute, the locomotion duration decreases abruptly and remains constant (2.5 s). Before the 55^th^ minute, *agn^ts3^*is characterized by the same locomotion duration as that of *CS*. The differences start being observed only on the 60^th^ minute. In a series of points (5–15, 25, and 35 min), the locomotion duration in *agn^ts3^*is statistically significantly higher than that in *Berlin*. No significant differences from *Or-R *were detected. In the beginning of the experiment,* CS *larvae have the highest speed of motion (~ 0.45 mm/s), which decreases appreciably rapidly and reaches 0.4 mm/s by the end of the experiment.* Berlin *is originally characterized by a lower speed of motion compared with *CS *(~ 0.36 mm/s). However, in this case the speed of motion does not decrease; instead, it slightly increases by the end of the experiment. Hence, starting with the 40^th^ minute of the experiment, the differences between *Berlin *and *CS *disappear. *Or-R* is characterized by a lower speed of motion compared with *CS *throughout the entire experiment (0.38 mm/s in the beginning and ~ 0.34 mm/s by the end of the experiment). Statistically significant differences from* Berlin *were detected on the 50^th^ and 60^th^ minutes. The total speed of locomotion in *Berlin *and *Or-R *differs statistically significantly throughout the entire experiment.* agn^ts3^*is characterized by a high speed of locomotion. Between the 10^th^ and 50^th^ minutes, no differences with *CS *are observed. Differences with *Berlin *(55–15, 25–35, 60) and *Or-R *(10–50) are observed for a series of points.

Thus, examination of the temporal dynamics of locomotor activity parameters demonstrated that the activity index of larvae and the locomotion frequency coincide for the series *CS → Berlin → Or-R → agn^ts3^*. The activity index in *Berlin *remains low during the entire experiment, while *Or-R *is characterized by a very rapid drop in the originally high activity index.* Or-R *exhibits the largest difference for the locomotion duration, while *agn^ts3^*is closer to the wild-type stock. This cross-alteration of locomotion duration and frequency presumably leads to the absence of differences in the activity index between *Berlin*, *Or-R, *and* agn^ts3^*. The speed of locomotion decreases in the series* CS *→ *agn^ts3^*→ *Berlin *→ *Or-R*.

**Distribution over the shape of movement trajectories (tracks)**

**Fig. 3:**
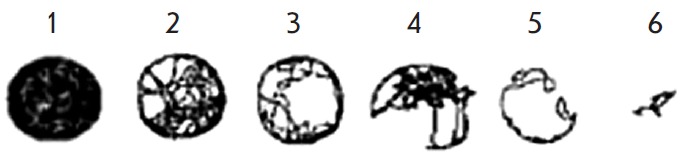
Examples of track classes. 1 – complete coverage of all space available for movement in the experimental camera; 2 – insignificant defects of coverage; 3 – significant defects of coverage; 4 – distorted movement in space; 5 – strong defects of spatial movements; 6 – dramatic defects of movement in space

The visuospatial orientation ability (i.e., the ability of an animal to orient in the environment when performing exploration and the way an animal stops it with time) can be characterized by analyzing tracks (motion trajectories). All the tracks analyzed were subdivided into six classes. The characteristic shape of each class of tracks is shown in *Fig. 3*.

**Fig. 4:**
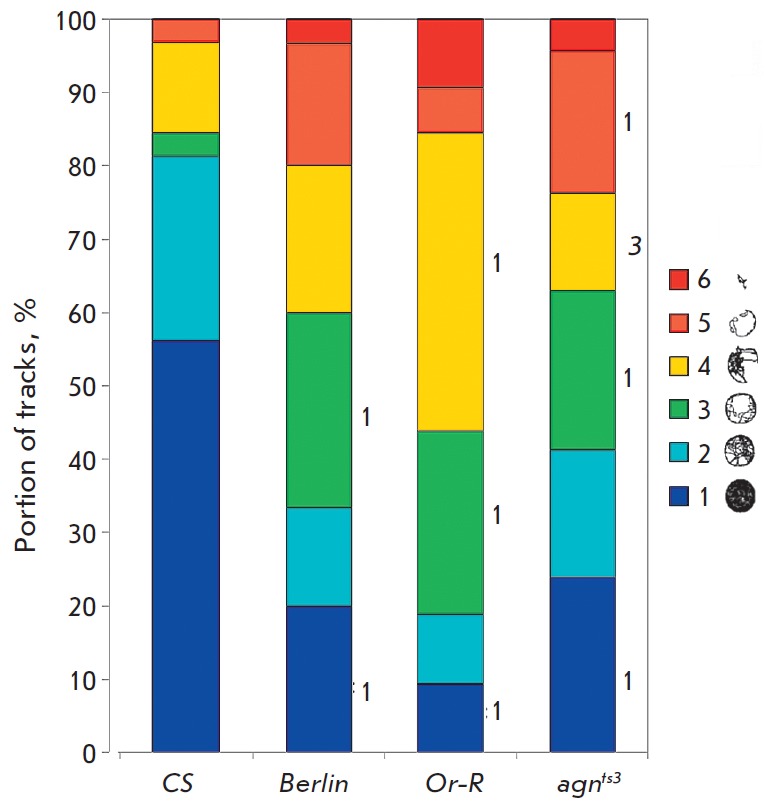
Comparison of distributions of track classes. * – significant difference from *CS*, *p * < 0.05; 1 – significant difference from *CS*, 2 – significant difference from *Berlin*, 3 – *agnts3 *significant difference from *Or-R*. Paired Student’s* t*-test for shares, *p * < 0.05

The distribution of larval tracks over classes has significant interlinear differences. The decrease in the number of class *1 *tracks was most noticeable (~ threefold) in *Berlin*, *Or-R, *and *agn^ts3^*as compared to *CS* *(Fig. 4)*. The number of class *3* and *6* tracks increases statistically significantly in *Berlin*.

In *Or-R*, only the number of class *4 *tracks increases reliably. A decreased number of class *2 *tracks and a noticeable increase in the number of class *3 *tracks is observed for *agn^ts3^*.

Thus, each stock has its own defects of locomotor behavior. *Berlin *is characterized by a reduced activity index and disturbed track distribution. *Or-R *has a spontaneous activity defect: a rapid decrease in the activity index, while the speed of motion is retained. All these disturbances may attest to the significant defects in visuospacial orientation that are observed in *Berlin*,* Or-R, *and *agn^ts3^*.

**Studying the locomotor activity of imago and its contribution to cognitive abilities**

**Fig. 5:**
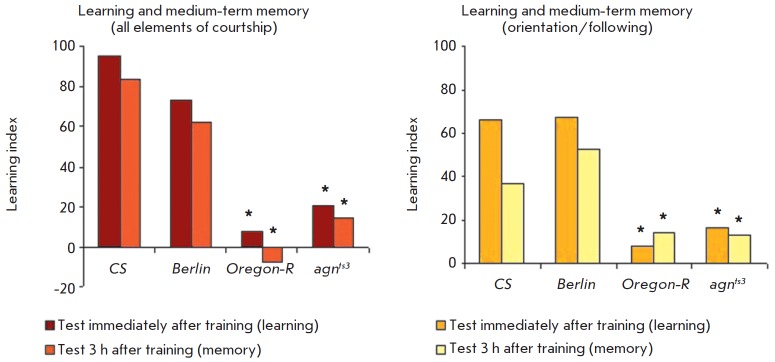
Contribution of defects in orientation and following to learning and memory disturbances. * – significant difference from *CS*, *p * < 0.05, two-sided randomization test

Recording the behavior under conditioned reflex suppression of courtship allows one not only to calculate the learning index (LI) with allowance for all the elements of non-sexual (locomotor activity, preening, rest) and sexual behavior (orientation/following, wing vibration, tapping, licking, copulation attempt), but also to analyze the recorded behavioral ethograms individually for each parameter. *Figure 5* demonstrates that, as it was shown earlier, the learning processes and formation of medium-term memory (3 h) are dramatically disturbed both in *agn^ts3^*mutants [8] and in *Oregon-R* males [13].

If one calculates the learning index with allowance for a random element of sexual behavior, it will be possible to determine why the learning and memorization defects develop. It turned out that suppression of orientation/ following makes the key contribution to the total suppression of courtship. In terms of this parameter,* Oregon-R *and *agn^ts3^*males were unable to learn: the conditioned reflex suppression of courtship was not developed immediately after training. Three hours after the training, the learning index remained at the same level; it also differed statistically significantly from the LI in *Canton-S*. It is clear from *Fig. 5* that learning and memory defects in *Oregon-R *and *agnts3 *can be explained by a disturbed orientation/following behavior.

**Fig. 6:**
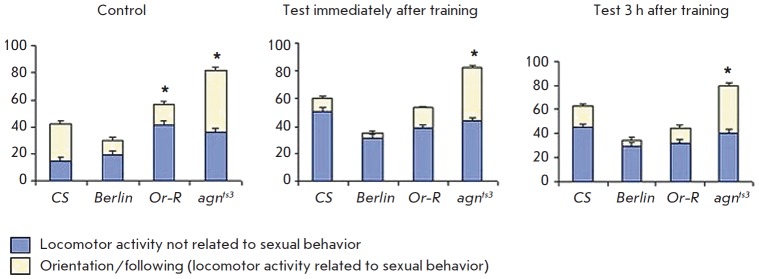
Portions of locomotor activity connected and not connected to sexual behavior immediately and 3 h after training. * – significant difference from *CS*, αR ≤ 0.05, one-sided randomization test

Let us discuss these processes more thoroughly, with a focus on such a parameter of locomotor behavior of imago during intrapair interactions as the locomotor activity of males not related to sexual behavior. *Figure 6* shows that the locomotor activity in the control (naive* Oregon-R *and *agn^ts3^*males) is statistically significantly higher than that in the wild-type *CS *stock. The activity levels were comparable with those in *CS *males immediately and 3 h after training.

As for the activity related to sexual behavior – orientation/ following (intrapair interaction between a male and a female) – it is twice as low in *Berlin *and *Oregon- R *males but twice as high as that in wild-type *agn^ts3^* males. This form of activity is expected to decrease abruptly after training, when conditioned reflex suppression of courtship is observed (an abrupt decrease in the share of activity related to sexual behavior and an abrupt increase in the share of usual locomotor activity). This actually is observed immediately after training in *CS*, *Berlin, *and *Oregon-R *males but not in mutant* agn^ts3^*males, in whom the activity related to sexual behavior is fourfold higher than that in *CS *males, thus being indicative of a learning defect. Three hours after the training, when the orientation/following parameters may slightly decrease (like in *CS*), orientation/ following in *agn^ts3^*fruit flies is twice as high as that of* CS*. The contribution of this component to the conventionally calculated courtship indices in the wild-type* CS *stock is 35% (learning) and 42% (memory); in the wild-type *Berlin *stock, 33% (learning) and 34% (memory); in the wild-type *Oregon-R *stock, 50% (learning) and 40% (memory); and in *agn^ts3^*mutant, 85% (learning) and 83% (memory).

Thus, each stock is characterized by a typical ratio between two activity forms. This seems to provide a good opportunity to test the diagnostic symptom belonging to the triad of cognitive impairments in Williams syndrome: unusually strong gaze fixation on faces (in this case on a target courtship object). Furthermore, this analysis method allows one to take into account the learning component (individual experience). Let us remind the reader that in patients with Williams syndrome, the unusually strong hypersocialization (gaze fixation on strangers’ faces) does not lead to tight contacts with peers, school friends, or making friends. In other words, a patient constantly addresses the same stimulus (the face), without properly interpreting the response.

The findings provide grounds for claiming that cognitive defects caused by insufficient suppression of orientation/ following of a partner were found in *Oregon- R *and *agn^ts3^*males. The orientation defects manifest themselves as early as in larvae; male larvae exploring the environment demonstrate abrupt disturbance of motion trajectories in space and temporal dynamics of the activity index, and the locomotion frequency of larvae.

## CONCLUSIONS

*Drosophila melanogaster *stocks carrying different variants of the *agnostic *locus with changes in the regulatory and structural regions of the gene encoding LIM kinase 1 (LIMK1) were used to simulate the human deletion Williams syndrome. It is believed that hemizygosity for the *limk1 *gene results in disturbance of motor functions, cognitive defects of visuospatial orientation, and strong gaze fixation on a partner [2].

Simulation of this syndrome with the involvement of mutant and spontaneous variants of the *agnostic* locus made it possible to reveal the effect of changes in the *limk1 *gene structure on locomotor and cognitive manifestations, including changes in locomotor activity parameters in larvae and abrupt changes in tracks in *Oregon-R *and *agn^ts3^*males during spatial orientation. Extensive training and memorization defects under conditioned reflex suppression of courtship are observed in imagoes of the same lines due to an abruptly increased orientation towards a partner and following it.

Based on the data obtained by sequencing the* limk1 *gene from *Canton-S*, *Berlin*, *Oregon-R*, and* agn^ts3^*stocks (the International Database of Genetic Data GenBank (http://www.ncbi.nlm.nih.gov/Genbank/, numbers Dlimk1_allforGenbank.asn.1 dmellimk1- CantonS JX987486 Dlimk1_allforGenbank. asn.1 dmel-limk1-agnosticts3 JX987487; Dlimk1_allforGenbank. asn.1 dmel-limk1-Oregon-R JX987488; Dlimk1_allforGenbank.asn.1 dmel-limk1-Berlin JX987489; Dlimk1_allforGenbank.asn.1 dmel-limk1- CantonS JX987486; Dlimk1_allforGenbank.asn.1 dmel-limk1-agnosticts3 JX987487; Dlimk1_allfor- Genbank.asn.1 dmel-limk1-Oregon-R JX987488; Dlimk1_allforGenbank.asn.1 dmel-limk1-Berlin JX987489)), one can assume that the defect of the LIM and PDZ domains in *Oregon-R *stock fruit flies is accompanied by changes in the locomotor behavior and abrupt cognitive impairment. The changes in the LIM and PDZ domains of LIMK1 also reduce both the visuospatial orientation and learning abilities. An insertion of S-transposon in the 3’-untranscribed region of the *limk1 *gene in *agn^ts3^*mutant also results in rather interesting outcomes. Mutant *agn^ts3^*had defects in orientation ability and a significant impairment of the cognitive sphere, which was accompanied by LIMK1 hyperexpression [8, 14].

It should be mentioned that the results of our study lay the groundwork for developing a method for the rapid assessment of the effect of various pharmacological agents on the locomotor and cognitive ability of* Drosophila*. The proposed methods for recording the behavior of *Drosophila *larvae and imagoes can be used to search for drugs correcting locomotor and cognitive impairments. The celerity and relatively low cost of studies using *Drosophila *melanogaster make it a virtually ideal object for preliminary experimental testing of therapeutic drugs. The drugs that have passed through this stage can be moved to the next stage of testing using vertebrates, which are closer to humans.

The revealed relationship between mutational damage to the *agnostic *gene and defects in the locomotor and cognitive spheres make it possible to use this model to study genome diseases, and Williams syndrome in particular.

## Abbreviations

CRSC: conditioned reflex suppression of courtship
LI: learning index
CI: courtship index
